# Hyperconjugation in Carbocations, a BLW Study with DFT approximation

**DOI:** 10.3389/fchem.2013.00037

**Published:** 2014-01-07

**Authors:** Zakaria Alamiddine, Stéphane Humbel

**Affiliations:** Centrale Marseille, Aix Marseille Université, CNRS, iSm2 UMR 7313Marseille, France

**Keywords:** carbocation, valence bond, silicon, hyperconjugation, conjugation

## Abstract

The geometry of ethyl cation is discussed, and the hyperconjugation effect in carbocations is evaluated at the B3LYP/6-311G(d) level. The Block Localized Wavefunction (BLW) method is used for all evaluations of the hyperconjugation, considered as the energy gained by the delocalization onto the C^+^ atom. This energy is defined as the energy difference between the delocalized (standard) calculation, where the electrons are freely delocalized, and a localized form where the positive charge sits on the carbon center. It is evaluated for 18 carbocations, including conjugated systems. In these cases we were particularly interested in the additional stabilization brought by hyperconjugative effects. Among other effects, the β-silicon effect is computed. Hyperconjugation amounts in several cases to an energy similar to conjugation effects.

## Introduction

Carbocations' stabilization by hyperconjugation is one of the cornerstones of chemistry, and has received a considerable attention, particularly in educational, organic, and theoretical literature (Hehre, 1975). They are involved in numerous reactions, whenever an anionic chemical group leaves a carbon atom, as it is the case in S_N_1 reaction for instance, or by positively charged species attachment (White et al., 1999). Olah et al. have boosted their experimental study with exceptionally strong acids (Olah, 1993, 2001). However, computational studies are essential for the evaluation of the energetics at work (Lambert and Ciro, 1996; Müller et al., 2005). The recent review by Aue (2011) made a special emphasis on the study of their stability, plus a presentation of carbocations of practical interest, for instance in biological systems.

Carbocations stability is a key in numerous reaction mechanisms, particularly near transition states, where bond breaking/forming processes modify the electronic density of a species. Thus, their stabilization occurs frequently in systems that can be distorted from their equilibrium geometry. Their stability relies particularly on charge delocalization over the whole chemical species, and this can be attained via conjugation and hyperconjugation (Müller et al., 2005; Hadzic et al., 2011; Newhouse and Baran, 2011; Emanuelsson et al., 2013; Zimmerman and Weinhold, 2013). Even when it is small in magnitude, hyperconjugation can determine reactivity, and is of primary importance (Cieplak, 1999; Ingold and DiLabio, 2006; Braïda et al., 2009; Fernandez et al., 2013).

The conformation plays an important role, and sometimes it can be used to switch the delocalization off (deconjugated bond), and evaluate its effects by difference with the conjugated conformation (Wiberg et al., 1990; Gobbi and Frenking, 1994).

**Scheme 1:**
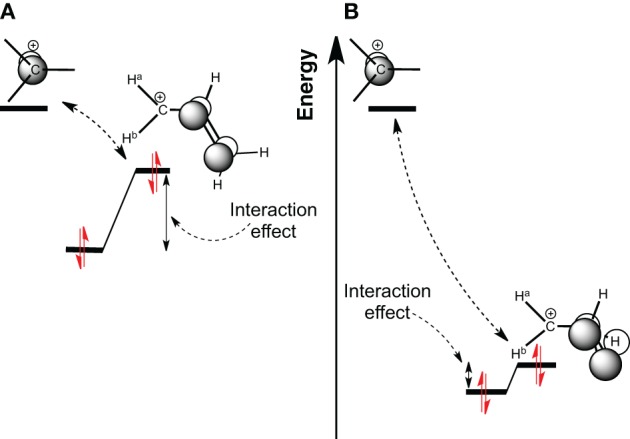
**Molecular Orbital diagram of a carbocation 2p orbital, interacting with (A) a π-CC bonding orbital; (B) a π-CH bonding orbital**.

Conjugation involves an interaction between π orbitals. It reputedly implies large stabilization energy (or resonance energy) and the effect extends across several bonds (Milian-Medina and Gierschner, 2012). Here, in the carbocation case, the charge delocalization corresponds to the interaction between the empty π orbital of the carbocation center, and at least one filled π orbital expanded on neighboring atoms (Scheme 1A) (Alabugin et al., 2011). We shall use the allyl cation as a model system to evaluate such stabilization. The hyperconjugation (Scheme 1B) involves filled CH orbitals, which are in principle lower in energy. Because orbitals interact better if they are close in energy, the effect is in principle larger for conjugation than for hyperconjugation.

**Scheme 2:**
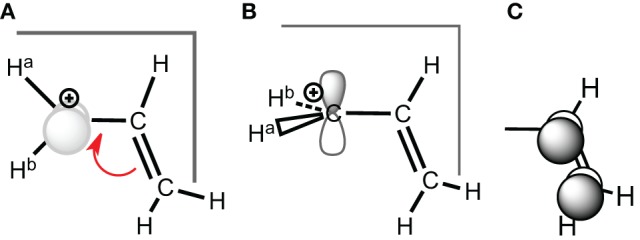
**(A)** Conjugated carbocation for the allyl cation; **(B)** deconjugated case; **(C)** corresponding conjugated **π**-orbital.

**Figure 1:**
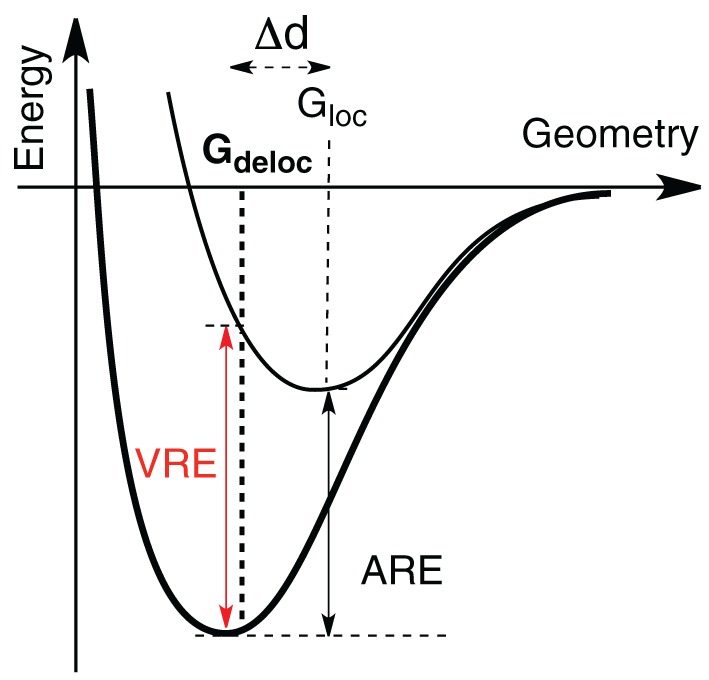
**Vertical (VRE) and Adiabatic (ARE) Resonance Energy**. In bold is the energy curve of the delocalized wave function, while in plain is the localized. VRE and ARE differ by the geometry used to compute the localized wave function. The optimum geometry with the delocalized wave function is G_deloc_, and G_loc_ for the localized one.

As stated above in the allyl cation, a deconjugation by rotation around a CC bond (Scheme 2) can give an estimation of the resonance energy. However, this gives an underestimation of the energy because hyperconjugative effects lower the rotated structure (Mo, 2004). The value of the resonance energy in allyls has been the subject of some debates, which chiefly concerned the allyl anion (Mo et al., 1996; Mo and Peyerimhoff, 1998; Barbour and Karty, 2004; Linares et al., 2008). As far as the cation is concerned, there are less discrepancies among the authors although electronic correlation is significant and some variations are encountered. We shall retain that, with an Orbital Deletion Procedure (ODP) (Mo, 2006) the resonance energy in the allyl cation was evaluated to 36.6 kcal/mol at the HF level, and to 48.8 with B3LYP/6-311+G(d,p) level using the “Block Localized Wavefunction” (BLW) approach (Mo et al., 2007). These correspond to “Adiabatic Resonance Energies” (ARE), which means that geometrical parameters are relaxed in the localized calculation (Figure 1). The later value is close to the value of 50 kcal/mol, obtained with a corrected Hydride Ion Affinities procedure (HIA) (Aue, 2011). It is also close to the value obtained with our Lewis-based Valence Bond BOND scheme, 55 kcal/mol (Linares et al., 2006), although our value is to be considered as a Vertical Resonance Energy (VRE) because the geometry of the localized structure is constrained to that of the delocalized system.

As reminded above, hyperconjugation is frequently considered as a (small) second order conjugation, which is justified by the lower energy of CH bonding orbitals compared to π (Scheme 1). This is particularly true in neutral systems, and the interaction can be small in these cases. However, there are evidences that hyperconjugation can be large, and even of similar magnitude as conjugation (Daudey et al., 1980; Mullins, 2012; Wu and Schleyer, 2013). Large hyperconjugation effects (in silicon substituted species) are reported to lead to very significant rate enhancements (up to 10^12^ times larger) (Lambert and Chelius, 1990; Creary and Kochly, 2009). They also have been isolated and an X-ray structure is even available.^1^

Several approaches are being used to describe the conjugation and hyperconjugation effects. We reminded in the introduction different publications using isodesmic reactions, based on hydride abstraction. Hyperconjugation in neutral systems has also been described by a large panel of methods such as the Energy Decomposition Analysis (EDA) (Fernandez and Frenking, 2006; Feixas et al., 2011; Mo et al., 2011), Valence Bond (VB) (Shaik and Hiberty, 2008; Braïda et al., 2009; Havenith and Van Lenthe, 2009) or the already mentioned BLW (Bickelhaupt and Baerends, 2003; Mo et al., 2004a,b; Mo and Schleyer, 2006; Wu et al., 2012), and the well-known Natural Bond Orbital analysis (NBO) (Weinhold, 2003; Glendening et al., 2012).

There is also a rich literature on silicon substituted carbocations, and cross method evaluations have been recently published on these systems (Fernandez and Frenking, 2007; Dabbagh et al., 2012, 2013). Still on carbocations, Schleyer et al. very recently (Wu and Schleyer, 2013), showed large hyperconjugation effects in various strained systems. However, for simple carbocations, which are our subject here, there have been fewer studies. Particularly, the values published by Mo (2006) with the BLW method at the HF level have not been updated with a care for electronic correlation.

Such an evaluation at the correlated level is certainly desired, and this is an objective of the present paper. We evaluated the energetics of hyperconjugation at the B3LYP/6-311G(d) level, which includes some correlation effects. We used the BLW approach in all the cases. Despite some discussions on its apparent basis set dependency (Mo et al., 2010; Zielinski et al., 2010) this type of calculation is becoming a standard for such an evaluation (Steinmann et al., 2011; Wu et al., 2012; Fernandez et al., 2013). Our BLW results shall update and extend the values published previously at the uncorrelated level (Mo, 2006). We expect that, as it was the case for conjugation, correlated value for hyperconjugation will be somehow larger than Hartree-Fock.

The paper is organized as follows. In the computational considerations, we first define our levels of calculations, programs we used, and we write a short memo on the way we used the “BLW” approach in the specific case of carbocations. We then turn our attention to the conformations of the ethyl cation as a model of all the hyperconjugated cations. The results and discussion part is divided into three subsections. The first one deals with the hyperconjugation in the ethyl cation. The second extends to secondary and tertiary carbocations, with methyl substituents and silicon β-effects. In the last part, we added a conjugated link between the C^+^ atom and the substituent (e.g., a C#C triple bond). We evaluated here the incremental stabilization due to hyperconjugation in already conjugated species.

## Computational considerations

The computations of the ethyl cation displayed in Table 1 were done with Gaussian 03 (Frisch et al., 2004). The three methods (HF, B3LYP, and CCSD) were used for the geometrical optimization with two basis sets, Pople's 6-311G(d) (Krishnan et al., 1980) and Dunning's cc-pvQZ (McLean and Chandler, 1980). DFT calculations are not very sensitive to the size of the basis set, but CCSD is much more basis set dependant. For the B3LYP calculations we used the default implementation of Gamess, with the original VWN5 correlation functional^2^ rather than the defaults Gaussian's implementation (Vosko et al., 1980; Lee et al., 1988; Becke, 1993).^3^ As it is also the default in Gamess, 6D orbitals were used throughout. As the basis set dependency was small only the 6-311G(d) results are discussed here. The results obtained with the cc-pvQZ basis set are given in the supplementary materials.

**Table 1:** **Energetics and key geometrical parameters of the ethyl cation in the conformations of Figure 2 (ΔE in kcal/mol, d_CC^+^_ in Å, ∠_CCH_ in °)**.

**Conformation**	**(1a)**	**(1b)**	**(1c)**
**6-311G(d)**			
**HF**			
ΔE	0.0	−0.8	−0.6
d_CC^+^_	1.438	1.427	1.373
∠_CCH_	115 (107)	95	58
**CCSD**			
ΔE	0.0	x	−5.0
d_CC^+^_	1.425	x	1.386
∠_CCH_	117 (107)	x	58
**B3LYP**			
ΔE	0.0	x	−3.0
d_CC^+^_	1.412	x	1.380
∠_CCH_	118 (108)	x	58

*For the conformation (1a), the second angle (added in parenthesis) concerns the CCH angle for the out of plane hydrogens*.

For all the BLW calculations, we used a version of Gamess that was modified by Mo to implement the BLW method (Mo and Peyerimhoff, 1998; Mo et al., 2000; Cembran et al., 2009). This implementation permits to re-optimize the geometry of the cations with the localization constraints. This feature was used to compute the geometrical effects of the localization: we obtained the CC^+^ bond lengthening, and its impact on the resonance energy (hence we obtained both VREs and AREs). However, the ARE will be of little use here. We rather discuss the vertical energies because they can concern directly and unequivocally reactive intermediates in their genuine geometry. Geometrical variations upon localization/delocalization (Δd) are discussed though.

With BLW, we used there the standard 6-311G(d) basis set, with no diffuses on heavy atoms and no polarizations on hydrogens. These restrictions intend to preserve the localized calculations' meaning. Diffuse orbitals on first neighbors of the carbocation, as well as π orbitals on H atoms could bring some confusion on the validity of the localization constraints (Galbraith et al., 2013). In these calculations we oriented the systems in such a way that the π system is along the z axis, and two blocks are defined for the localized calculations. One contains zero electrons (it is empty), and is defined over the p_*z*_, d_*xz*_, d_*yz*_ atomic orbitals of the C^+^ site. This block ensures an appropriate localization of the positive charge. The other block contains all the electrons and is defined over all the remaining orbitals. For the delocalized calculations, we removed the (empty) block, and added the p_*z*_, d_*xz*_, d_*yz*_ atomic orbitals to the other block, so delocalization is now allowed.

The analysis of the BLW results concerns both energies and difference between electronic densities. For these densities we used two “cube” files generated by Gaussian 03. One has densities obtained with the orbitals of the localized calculation. The second uses the delocalized orbitals.^4^ The density differences at each point of the grid defined in the cube files were drawn with the VMD freeware.^5^ We refer to these drawings as Electronic Densities Difference maps (EDD maps). They indicate the flux of electron density (gain/loss) when localization constraints are relaxed.

### Ethyl carbocation: on the Cs geometry

**Scheme 3:**
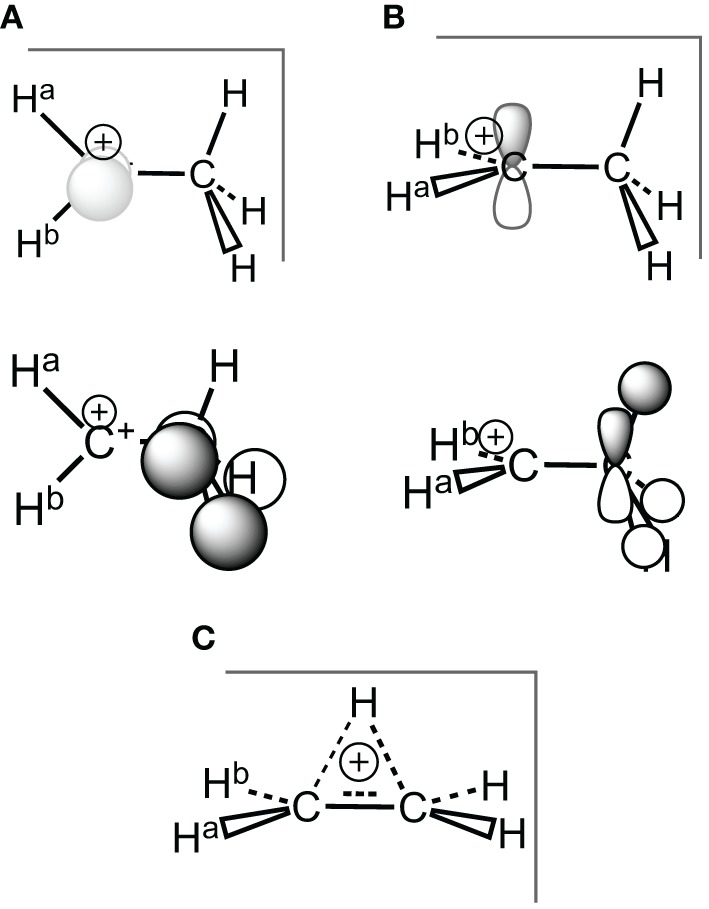
**Ethyl carbocation, conformations and corresponding hyperconjugated orbitals. (A)** Cs conformation 1a with hyperconjugation from a π-CH bonding orbital; **(B)** Cs conformation 1b with hyperconjugation from a σ-CH bonding orbital; **(C)** C_2*v*_ H^+^ bridged conformation 1c.

**Figure 2:**
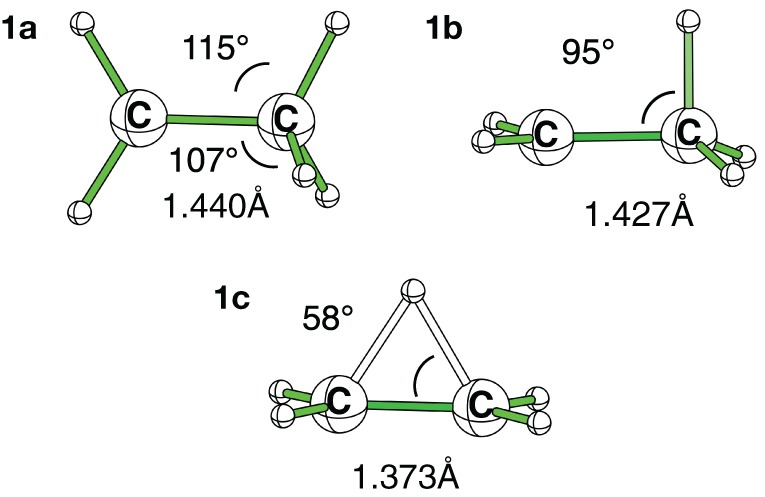
**HF/6-311G(d) optimized geometries for the three conformations of the ethyl cation**.

It is noteworthy that in the ethyl cation, which is the smallest system useful to describe and evaluate the hyperconjugation effects, the conformation with an hyperconjugation from σ-CH bonding orbital (Scheme 3B) is a minimum at the Hartree-Fock level, but this minimum collapses to a bridged conformation (Scheme 3C) at correlated levels of calculation such as B3LYP, and CCSD for both 6-311G(d) and cc-pvQZ basis sets. The corresponding HF/6-311G(d) optimized geometries are displayed in Figure 2 and both energetics and geometrical values are in Table 1. The results with the cc-pvQZ basis set are given in the supplementary materials.

Because it involves bond breaking/forming, the bridged cation (1c) needs *a priori* a higher level of computation than the hyperconjugated system (1a) (van Alem et al., 1998). However, all the correlated levels converged to similar energy differences, within a few kcal/mol. The average energy difference between the two conformations is ΔE_ac_ = −4 ± 2 kcal/mol. This is one order of magnitude smaller that the hyperconjugation energies at work (*vide infra*).

At the HF/6-311G(d) level the (1b) conformation and the bridged one (1c) are two different minima, but the small ∠_CCH_ angle (95°) indicates that the proton transfer has already started in (1b), and is effective in (1c). Such 1,2 transfers are related to chemical reactivity (Crone and Kirsch, 2008) (bonds are changing) rather than hyperconjugation itself. However, the limit between reactivity and resonance is somehow difficult to define in hyperconjugation because the orbitals that act as donors are C-H bonding orbitals, hence single bonds are partly broken, which corresponds (partly) to a chemical reaction. For a fair and transferable/comparable evaluation of the hyperconjugation effects, we decided to use the (1a) conformation, even though it is characterized as a transition state. The fact that at the correlated levels (CCSD and B3LYP) conformation (1b) collapses to (1c) has also motivated our choice. The (1a) conformation corresponds to the interaction between a π-CH bonding (filled) orbital and the pure empty *p* orbital of the carbocation (Scheme 3A). A similar scheme can be drawn for conformation (1b). The hyperconjugation in conformation (1a) is shown by both the CC^+^ distance, which is shorter than a normal single bond, and the out of plane CCH angle which is smaller than normal sp^3^ angles (e.g., at the B3LYP/6-311G(d) level d_CC^+^_ = 1.412Å and ∠_CCH_ = 108°—Table 1). The results are similar with the cc-pvQZ basis (see Supplementaries, Table S1).

## Results and discussion

**Figure 3:**
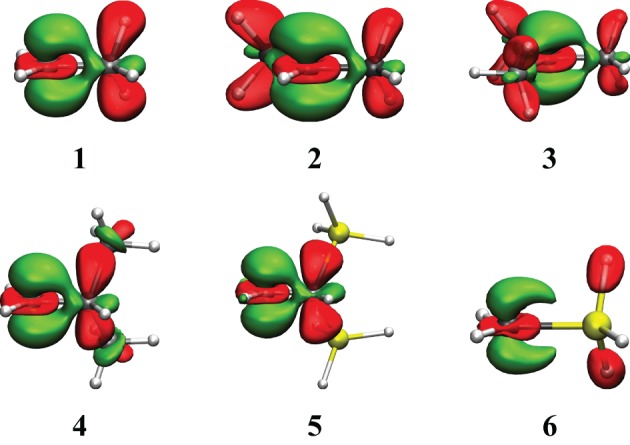
**Electron Density Difference maps (EDD) for the hyperconjugated carbocations reported in Table 2**. In green is the electron gain, and in translucent red is the electron loss. An isodensity of 4·10^−3^ was used throughout.

**Figure 4:**
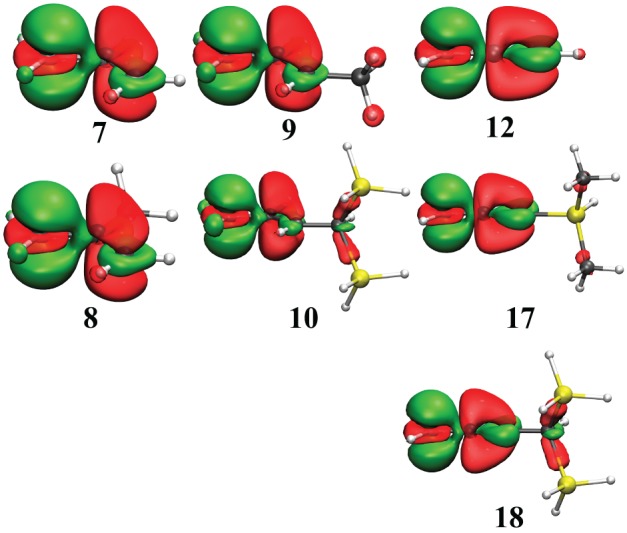
**Electron Density Difference map (EDD) of key conjugated carbocations from Table 3**. In green is the electron gain, and in translucent red is the electron loss. An isodensity of 4.10^−3^ was used throughout.

The results for hyperconjugation in simple carbocations are in Table 2 and Figure 3, while Table 3 and Figure 4 concern the evaluation of the hyperconjugation in conjugated carbocations. In the tables, the two first columns correspond to VRE and ARE as defined in Figure 1. In the three last columns is the CC^+^ bond variation when hyperconjugation is activated. The CC^+^ bond shortens when the delocalization is allowed and Δd is thus always negative. These results were of course expected since the electronic delocalization evidently builds a kind of π bonding between these two atoms. In the discussions that follow, VRE's are used more often than ARE's because their definition is more straightforward.

**Table 2:** **Hyperconjugated carbocations: vertical and Adiabatic resonance energy (VRE and ARE) for the hyperconjugated systems**.

**Species**		**VRE**	**ARE**	**d_CC_^+^(deloc)**	**d_CC_^+^(loc)**	**Δ*d*^a^**	
1^b^	CH_3_-CH_2^+^_	19.6	12.3	1.438	1.510	−0.07	
1	CH_3_-CH_2^+^_	29.7	23.2	1.412	1.513	−0.10	
2	(CH_3_)_2_−CH^+^	39.9	34.4	1.443	1.513	−0.07	
3	(CH_3_)_3_−C^+^	45.0	40.7	1.463	1.517	−0.05	
4	*^i^*Pr-CH_2^+^_	33.0	25.6	1.409	1.529	−0.12	
5*^c^*	DSM-CH_2^+^_	61.8	51.3	1.371	1.493	−0.12	
6	SiH_3_-CH_2^+^_	12.6	9.4	1.894	2.013	−0.12*^d^*	
7	H_2_C=CH−CH_2^+^_	56.0	49.1	1.381	1.498	−0.12	

*B3LYP/6-311G(d) level is used, unless specified. All the structures are Cs symmetric and resemble to structure (a) of the ethyl cation—Scheme 3*.

a *Δ*d* = d_CC^+^_(deloc)–d_CC^+^_(loc)*.

b *HF/6-311G(d) level*.

c *DSM = −CH(SiH_3_)_2_*.

d *Si-C^+^ bond*.

**Table 3:** **Vertical and adiabatic resonance energy (VRE and ARE) for the conjugated systems**.

**Species**		**VRE**	**ARE**	***d*_CC_^+^(deloc)**	**d_CC_^+^(loc)**	**Δ*d*^a^**
7	H_2_C=CH–CH_2^+^_	56.0	49.1	1.381	1.498	−0.12
8	CH_3_-2-Allyl^+^	57.4	50.0	1.389	1.510	−0.12
9	CH_3_-3-Allyl^+^	66.9	58.8	1.368	1.487	−0.10
10	DSM-3-Allyl^+^	81.8	71.7	1.355	1.478	−0.12
11	C_6_H_5_–CH_2^+^_	68.7	61.2	1.368	1.488	−0.12
12	H-C#C–CH_2^+^_	54.1	49.0	1.345	1.434	−0.09
13	CH_3_-C#C–CH_2^+^_	63.3	54.8	1.336	1.414	−0.08
14	SiH_3_-C#C–CH_2^+^_	62.0	54.1	1.338	1.422	−0.08
15	*^t^*Bu-C#C–CH_2^+^_	67.9	58.1	1.333	1.406	−0.07
16^b^	TMS-C#C–CH_2^+^_	66.4	57.4	1.335	1.413	−0.08
17^c^	DMS-C#C–CH_2^+^_	65.1	59.0	1.336	1.427	−0.09
18^d^	DSM-C#C–CH_2^+^_	78.9	71.2	1.325	1.416	−0.09

*Calculations are at the same level as in Table 2*.

a *Δd = d_CC^+^_(deloc)–d_CC^+^_(loc)*.

b *TMS = −Si(Me)_3_*.

c *DMS = −SiH(Me)_2_*.

d *DSM = −CH(SiH_3_)_2_*.

To have in mind an order of magnitude for our calculations, we shall recall that for the allyl cation, the VRE amounts to 56.0 kcal/mol (Table 2, entry 7). This value is to be considered as large.

### Hyperconjugation in the ethyl cation

The HF geometries and energies obtained for the ethyl cation are similar to those obtained previously by Mo with the ODP procedure (Mo, 2006). With the B3LYP approximation there is a shortening of the CC^+^ bond, from 1.438Å at the HF level, down to 1.412Å. For the energetic values, we expected an increase at the correlated level, just as it was the case for allyl cation. In this case, Mo reported a resonance energy of 36.6 kcal/mol at the HF/6-311+G(d) level (Mo, 2006), and 48.8 kcal/mol with B3LYP/6-311+G(d,p)^6^ (Mo et al., 2007), which corresponds to 33% of increase. The relative variation is larger for the ethyl cation: the ARE varies here from 12.3 kcal/mol at the HF level (Table 2 entry 1) to 23.2 with B3LYP (entry 2). It corresponds to 90% of increase. The VRE amounts to nearly 30 kcal/mol. Hyperconjugation is thus smaller than conjugation in the allyl cation, but the order of magnitude is similar, with a ratio ethyl/allyl = 0.53.

NBO calculations^7^ on the ethyl cation give access to a second order perturbation theory analysis of the Fock matrix, where the hyperconjugation is evaluated to 18.2 kcal/mol for each of the two CH bonds concerned. The total hyperconjugation can thus be evaluated to 36 kcal/mol with this approach, which is slightly larger than our BLW evaluation. The electronic transfer from the CH bonds to the C^+^ atom amounts to 0.27 electron. For comparison, on the allyl cation NBO gives an interaction between the π bond and the C^+^ atom that amounts to 127 kcal/mol. Very logically, this electron transfer concerns 0.5 electron. With the NBO approach the ratio of the interactions is ethyl/allyl = 0.28. It is somehow smaller than with BLW. However, the perturbative evaluation of the interaction energy in the allyl might be subject to some caution due to the large effect we are addressing here perturbatively.

Both BLW and NBO evaluations of the hyperconjugation in the ethyl cation give a relatively strong hyperconjugative interaction, and this is consistent with the significant CC^+^ bond shortening, Δ*d* = −0.10 Å. We shall note that almost the same shortening is obtained in the allyl (Table 2, Δ*d* = −0.12 Å).

### Hyperconjugation and substitution effects

The substitution effects can be studied via two types of systems, depending on whether the substitution takes place on the carbocation atom, leading to secondary and tertiary carbocations, (cases **1**, **2**, **3**, **6** in Table 2) or if it takes place on the atom at the α-position (hence leading to β-substituted primary carbocations) (cases **4** and **5**).

The EDD map displayed in Figure 3 shows clearly the electron loss along the two CH bonds and the electron gain, with the shape of a π bond between the two carbon atoms. This corresponds to the idealized picture of hyperconjugation (Scheme 1). These EDD can only be used qualitatively, but very large differences can be visualized. For instance, the delocalization is obviously much larger in the ethyl cation (**1**) than in the SiH_3_ substituted equivalent (**6**). The computed energetics are consistent with the drawing: the VRE amounts to 29.7 kcal/mol in **1**, but is as small as 12.6 kcal/mol in **6**.

For cases **1**, **2**, **3**, not surprisingly, secondary and tertiary carbocations have larger and larger delocalization energy, up to VRE = 45 kcal/mol for the tertiary carbocation (CH_3_)_3_–C^+^. It is interesting to note that this value is similar to the conjugation in allyl.^8^ The effects of the methyl groups are not additive though. The first methyl brings about 30 kcal/mol, 10 for the second, 5 for the third. Hence, the average stabilization is 15 kcal/mol per methyl group. In (CH_3_)_3_C-C^+^, the three CC^+^ bond shortenings are accordingly smaller than in the ethyl cation, Δ*d* = −0.05Å although no direct correlation between bond shortening and hyperconjugation energy can be drawn. Steric effects may also be considered to moderate the bond shortening.

For cases **4** and **5**, the substitution with two methyls in α-position (**4**) gives almost no change in the delocalization energy as compared to ethyl cation. It is larger by only 3 kcal/mol (VRE = 33.0 kcal/mol). This variation is similar to the variation reported using other approaches, for instance by Aue with the Hydride ion affinity (+5 kcal/mol) (Aue, 2011). The di-silyl (SiH_3_)_2_ substitution (**5**) corresponds to a β-substituents, and leads to a significant increase in the resonance energy (by almost +30 kcal/mol). It is much larger than for the di-methyl (**4**) (CH_3_)_2_ moieties. The delocalization energy, VRE = 61.8 kcal/mol, is larger than the resonance energy in the allyl cation (**7**) at the same level.

Our results correspond roughly to Frenking's EDA evaluation of the relative stabilization energies between these two systems (33 kcal/mol) (Fernandez and Frenking, 2007), and similar results were reported by others for such silicon in β-position, for instance by isodesmic reactions^9^ (Lambert, 1990; Creary and Kochly, 2009). The delocalization energy *differences* are similar, but the delocalization energies differ, sometimes significantly. For instance, Frenking's EDA approach gives almost twice larger ΔE_π_ (100 kcal/mol for the di-silyl substitution) (Fernandez and Frenking, 2007).

One shall also note that the bond shortening for the CC^+^ bond is similar for these three primary carbocations, although the resonance energy can be very different. Although it is true that the CC^+^ distance variation reflects hyperconjugation, linear correlations cannot systematically be drawn.^10^ In **6**, the Si-C^+^ bond distance shortens by about −0.12Å, which is a large difference for such a small energetic effect. These SiC bonds are in principle longer and more flexible than CC bonds.^11^ The distance changes upon delocalization (Δ*d*) are probably less relevant than the actual bond length, obtained with standard calculations (that is without block localization constraints).

### Hyperconjugation in conjugated systems

We already mentioned that conjugation and hyperconjugation might have similar stabilization energies. We include in this part a few examples where conjugation is evaluated in typical systems such as the already discussed allyl cation, the aromatic benzyl cation and the C#C triple bond. These results are extended with some substituted systems to study how additional hyperconjugation operates in already conjugated systems. The results are in Table 3, with some EDD maps in Figure 4.

**Scheme 4:**
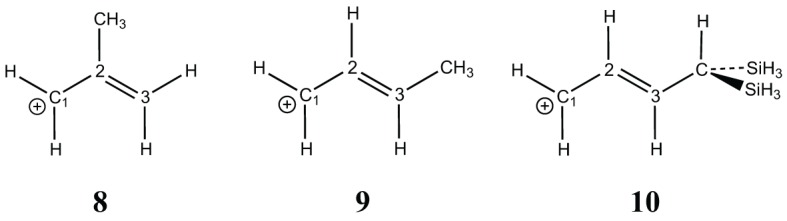
**Substituted allyl cations**.

The hyperconjugation effect on the allyl cation (**7**) is shown with three substitutions: one on position 2 (**8**) and the other two on position 3 (**9**, **10**) (Scheme 4). In **8** there is almost no effect: the resonance energy with the methyl substituent (57.4 kcal/mol) is very similar to the unsubstituted case (56.0 kcal/mol), but for a substituent in position 3, the resonance energy increases by about +10 kcal/mol (66.9 kcal/mol in **9**) with a methyl, and by +25 for the di-silyl methyl (81.8 kcal/mol in **10**). These hyperconjugated substituents act exactly as any conjugated sp^3^ substituent would act (e.g., −OH, −NH_2_). In **8** the methyl is conjugated with the double bond, but it is deconjugated from the positive charge on C_1_, hence its effect is negligible when delocalization is forbidden/allowed on C_1_. In **9**, the methyl is conjugated with both the double bond and with C_1_, hence the effect that we calculated on C_1_ is enhanced. It is even one of the largest resonance effects: it is similar to that of the benzyl cation (**11**), 68.7 kcal/mol.

The delocalization effects in the triply bonded systems are evaluated in the remaining systems (**12**–**18**). It is shown on the unsubstituted case that delocalization effects in the propyne cation (**12**) are similar to the allyl (**7**). In both species the resonance energy is evaluated to about 55 ± 1 kcal/mol. Substitutions at the acidic position in these systems increase the stabilization energies by about +10 kcal/mol for most species. The substitution by either a methyl (**13**) or a silyl (**14**) gives approximately the same resonance energy (about 63 kcal/mol). Larger substituents such as ter-Butyl (**15**), tri-Methyl Silyl (TMS) (**16**) or di-Methyl Silyl (DMS) (**17**) leads to only slightly larger resonance energies (65–68 kcal/mol). However, large resonance energy is found (again) with di-Silyl-Methyl (DSM) derivative. In that case, the (vertical) resonance energy increases to 78.9 kcal/mol. This value corresponds to an increment of some +25 kcal/mol (compared to the unsubstituted case), as it was the case for the allyl (**10**).

**Scheme 5:**
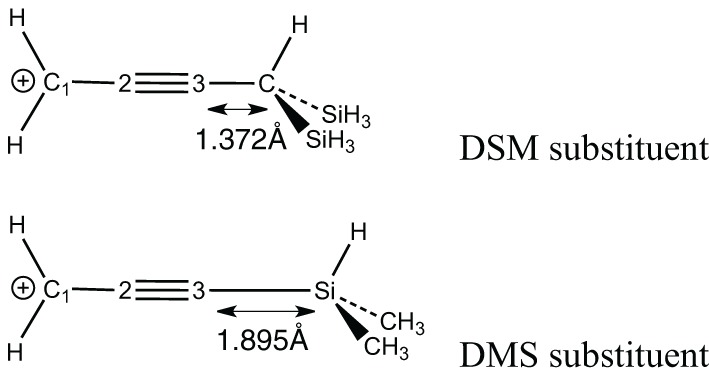
**Distance between the hyperconjugated substituent and the “unsaturated” system**.

The large resonance energy corresponds to more efficient σ-bond delocalization. However, in both DSM and DMS, the same type of SiC (or CSi) σ-bonds interacts with the conjugated carbocation. The stabilization increment is significantly larger for DSM (+25 kcal/mol) than for DMS (+10) we shall attribute it to the rather short distance between the conjugated link and the CSi bond in DSM (which is much smaller for DSM than DMS—Scheme 5). The interaction would finally be favored due to a better overlap.

Similarly to the previous series, EDD maps can be used to visualize main electronic effects in these conjugated systems (Figure 4). Of course, most of the delocalization comes from the conjugated link, but larger hyperconjugations correspond to larger domains of electron loss. This is the case for the cations **10** and **18** but much smaller domains appear for **9** and **17**.

## Conclusion

Using B3LYP we pinned down resonance energies in a variety of carbocations, with a special attention to hyperconjugative effects. Our discussion focused of vertical resonance energies, and we showed here how hyperconjugation could be of similar magnitude as conjugation, but this evaluation necessitates some correlated methods.

The fact that we considered cationic systems enhanced the delocalization effects. Smaller effects are expected (and reported) for neutral systems (Fernandez and Frenking, 2006). Nevertheless, a dimethyl-silyl substituent (DMS), delocalizes a significant amount of electron density from the CSi bonds onto the neighboring C^+^, and this hyperconjugation corresponds to a stabilization energy as large as 61.8 kcal/mol. This is to be compared to the vinyl delocalization onto the C^+^, in the allyl cation. It amounts to “only” 56.0 kcal/mol of conjugation; hence hyperconjugative effects on energy can be larger than conjugation. The CC^+^ bond distances are accordingly short, e.g., 1.371Å for the DMS-CH_2+_ carbocation.

Long-range hyperconjugative effects travel across an unsaturated linkage (a double or a triple bond here). We show that the energy associated to them can be as large as 25 kcal/mol. They can be logically extended to aryl linkages, for instance in para substituted benzyl cations.

## Supporting information available:

Cartesian coordinates of all the compounds discussed in the text.

### Conflict of interest statement

The authors declare that the research was conducted in the absence of any commercial or financial relationships that could be construed as a potential conflict of interest.

## Footnotes

^1^With two silicons and two tins in β position; see Schormann et al. (2002).

^2^Gamess (Version R2, 25 March 2010) from Iowa State University (Schmidt et al., 1993).

^3^The following keywords were used in Gaussian inputs: BV5LYP iop(3/76=1000002000) iop(3/77=0720008000) iop(3/78=0810010000).

^4^Orbitals are given as “cards” in the Gaussian input files. Of course, they were not re-optimized.

^5^VMD was developed by the Theoretical and Computational Biophysics Group in the Beckman Institute for Advanced Science and Technology at the University of Illinois at Urbana-Champaign (Humphrey et al., 1996).

^6^These are Adiabatic Resonance Energies (ARE). The value we report in Table 2 (49.1 kcal/mol) is slightly different from Mo's value due to the basis set difference: we used no diffuses on heavy atoms, and no p orbitals on the Hydrogens.

^7^NBO 5. Glendening, G. E. D., Badenhoop, J. K., Reed, A. E., Carpenter, J. E., Bohmann, J. A., Morales, C. M., et al. (Theoretical Chemistry Institute, University of Wisconsin, Madison, WI, 2004). Available online at: http://www.chem.wisc.edu/~nbo5

^8^The ordering is different from the one given by Alabugin et al. (2011). Our values are also systematically larger. We also note that the geometrical relaxation does not explain all the energy difference between Alabugin's and Mo's results (Mo, 2006): correlation effects are important as well.

^9^See for instance Lambert (1990).

^10^However, convincing linear regressions have been discussed on the subject, see Fernandez and Frenking (2007).

^11^ν_CC_= 995 cm^−1^ vs. ν_SiC_ = 700 cm^−1^, values extracted from http://webbook.nist.gov (a) ν_CC_ from Shimanouchi (1971) (b) ν_SiC_ from Shimanouchi (1977).
