# Supplementary material for: Hyperconjugation in Carbocations, a BLW Study with DFT approximation
Source: Front Chem. 2014 Jan 7;1:37. doi: 10.3389/fchem.2013.00037 (PMC3982569; doi:10.3389/fchem.2013.00037)
Supplement: Supplementary file 1 [file DataSheet1.PDF]

Hyperconjugation in Carbocations, a BLW Study with DFT approximation

*Zakaria Alamiddine[a,b] and Stéphane Humbel[*a]*

[a] Aix Marseille Université, Centrale Marseille, CNRS, iSm2 UMR 7313, 13397, Marseille, France

Fax: (+33) 4.91.28.82.34
E-mail: stephane.humbel@univ-amu.fr

[b] present address: Université de Nantes, CNRS, CEISAM, UMR 6230, 44322, Nantes, France

Supplementaries

Ethyl cation 3
- 6-311g(d) 4
- cc-pvQZ 5

Other systems (delocalized structures) 7
- 2 7
- 3 7
- 4 7
- 5 8
- 6 8
- 7 8
- 8 8
- 9 9
- 10 9
- 11 9
- 12 10
- 13 10
- 14 10
- 15 10
- 16 11
- 17 11
- 18 12

Ethyl cation

Table S1: Energetics and key geometrical parameters of the ethyl cation in the conformations of Figure 2 (ΔE in kcal/mol, d_{CC} in Å, $\angle_{CCH}$ in °). For the conformation (a), the second angle (added in parenthesis) concerns the CCH angle for the out of plane hydrogens).

	Conformation:	(a)	(b)	(c)
**6-311G(d)**	**HF**			
	ΔE	0.0	–0.8	–0.6
	dCC	1.438	1.427	1.373
	∠CCH	115 (107)	95	58
	**CCSD**			
	ΔE	0.0	x	–5.0
	dCC	1.425	x	1.386
	∠CCH	117 (107)	x	58
	**B3LYP**			
	ΔE	0.0	x	–3.0
	dCC	1.412	x	1.380
	∠CCH	118 (108)	x	58
**cc-pvQZ**	**HF**			
	ΔE	0.0	–1.1	–1.8
	dCC	1.433	1.413	1.370
	∠CCH	115 (107)	91	59
	**CCSD**			
	ΔE	0.0	x	–6.3
	dCC	1.414	x	1.377
	∠CCH	117 (106)	x	58
	**B3LYP**			
	ΔE	0.0	x	–4.0
	dCC	1.406	x	1.377
	∠CCH	118 (107)	x	58

6-311g(d)

(a)

HF

C	0.015127	0.760455	0.000000
H	-0.916490	1.306319	0.000000
H	0.919480	1.350864	0.000000
C	0.015127	-0.679376	0.000000
H	0.990595	-1.135221	0.000000
H	-0.587553	-1.004219	0.855862
H	-0.587553	-1.004219	-0.855862

B3LYP 5

C	0.022734	0.749678	0.000000
H	-0.916212	1.305662	0.000000
H	0.938664	1.343058	0.000000
C	0.022734	-0.662776	0.000000
H	0.985481	-1.165379	0.000000
H	-0.640372	-1.002377	0.831613
H	-0.640372	-1.002377	-0.831613

CCSD

C	0.020533	0.754001	0.000000
H	-0.923193	1.306295	0.000000
H	0.936721	1.351414	0.000000
C	0.020533	-0.671020	0.000000
H	0.993551	-1.156923	0.000000
H	-0.626735	-0.999337	0.845330
H	-0.626735	-0.999337	-0.845330

(b)

HF

C	0.030130	0.750233	0.000000
C	0.030130	-0.676758	0.000000
H	-1.083685	-0.789934	0.000000
H	-0.017862	1.314202	0.919501
H	-0.017862	1.314202	-0.919501
H	0.378927	-1.139659	-0.911142
H	0.378927	-1.139659	0.911142

(c)

HF

C	0.000000	0.686568	-0.062761
C	0.000000	-0.686568	-0.062761
H	0.924830	1.236746	-0.075278
H	0.924830	-1.236746	-0.075278
H	-0.924830	1.236746	-0.075278

H	-0.924830	-1.236746	-0.075278
H	0.000000	0.000000	1.054239

B3LYP 5

C	0.000000	0.690120	-0.064083
C	0.000000	-0.690120	-0.064083
H	0.933791	1.247685	-0.073183
H	0.933791	-1.247685	-0.073183
H	-0.933791	1.247685	-0.073183
H	-0.933791	-1.247685	-0.073183
H	0.000000	0.000000	1.061732

CCSD

C	0.063643	0.692930	0.000000
C	0.063643	-0.692877	0.000000
H	-1.050973	-0.000912	0.000000
H	0.071741	1.248664	0.936905
H	0.071741	1.248664	-0.936905
H	0.071886	-1.248366	-0.936844
H	0.071886	-1.248366	0.936844

cc-pvQZ

(a)

HF

C	0.016041	0.756323	0.000000
H	-0.916811	1.296820	0.000000
H	0.919098	1.345820	0.000000
C	0.016041	-0.677102	0.000000
H	0.988872	-1.133832	0.000000
H	-0.591828	-0.992066	0.855582
H	-0.591828	-0.992066	-0.855582

7

B3LYP 5

C	0.024037	0.745823	0.000000
H	-0.914605	1.296132	0.000000
H	0.938094	1.336805	0.000000
C	0.024037	-0.659985	0.000000
H	0.981977	-1.164824	0.000000
H	-0.646956	-0.991571	0.826633
H	-0.646956	-0.991571	-0.826633

CCSD

C	0.022136	0.747534	0.000000
H	-0.918429	1.291501	0.000000
H	0.933375	1.339974	0.000000

C	0.022136	-0.666304	0.000000
H	0.985123	-1.156662	0.000000
H	-0.632851	-0.981097	0.837368
H	-0.632851	-0.981097	-0.837368

(b)

HF

C	0.036902	0.739903	0.000000
C	0.036902	-0.673548	0.000000
H	-1.094769	-0.689210	0.000000
H	-0.019635	1.298642	0.920094
H	-0.019635	1.298642	-0.920094
H	0.345608	-1.153101	-0.913815
H	0.345608	-1.153101	0.913815

(c)

HF

C	0.000000	0.684823	-0.062735
C	0.000000	-0.684823	-0.062735
H	0.924118	1.233017	-0.076208
H	0.924118	-1.233017	-0.076208
H	-0.924118	1.233017	-0.076208
H	-0.924118	-1.233017	-0.076208
H	0.000000	0.000000	1.057659

B3LYP 5

C	0.000000	0.688267	-0.064096
C	0.000000	-0.688267	-0.064096
H	0.932309	1.243595	-0.072946
H	0.932309	-1.243595	-0.072946
H	-0.932309	1.243595	-0.072946
H	-0.932309	-1.243595	-0.072946
H	0.000000	0.000000	1.060928

CCSD

C	0.000000	0.688738	-0.064146
C	0.000000	-0.688738	-0.064146
H	0.932468	1.239543	-0.069613
H	0.932468	-1.239543	-0.069613
H	-0.932468	1.239543	-0.069613
H	-0.932468	-1.239543	-0.069613
H	0.000000	0.000000	1.048204

Other systems
(delocalized structures)

2

C	0.000000	0.000000	0.000000
C	-1.409410	0.000000	0.000000
H	-1.953830	-0.945450	0.000000
H	-2.017300	0.906270	0.000000
H	0.426380	1.002740	0.000000
C	0.483990	-0.811900	-1.284480
C	0.483990	-0.811900	1.284480
H	1.574210	-0.815840	-1.219650
H	1.574210	-0.815840	1.219650
H	0.181990	-0.311340	-2.202720
H	0.181990	-0.311340	2.202720
H	0.114670	-1.837060	-1.275940
H	0.114670	-1.837060	1.275940

3

C	0.00000	0.00000	0.00000
C	-1.46262	0.00000	0.00000
C	0.72885	1.26791	-0.00015
C	0.73170	-1.26547	0.00000
H	1.81267	1.17302	-0.00018
H	-1.91840	0.98789	-0.00004
H	0.10612	-2.15565	0.00008
H	0.39536	1.86641	0.86249
H	-1.81755	-0.58603	0.86278
H	1.41696	-1.27887	0.86271
H	0.39531	1.86626	-0.86286
H	-1.81757	-0.58610	-0.86272
H	1.41684	-1.27895	-0.86280

4

C	0.000000	0.000000	0.000000
C	-1.409410	0.000000	0.000000
H	-1.953830	-0.945450	0.000000
H	-2.017300	0.906270	0.000000
H	0.426380	1.002740	0.000000
C	0.483990	-0.811900	-1.284480
C	0.483990	-0.811900	1.284480
H	1.574210	-0.815840	-1.219650
H	1.574210	-0.815840	1.219650
H	0.181990	-0.311340	-2.202720
H	0.181990	-0.311340	2.202720

H	0.114670	-1.837060	-1.275940
H	0.114670	-1.837060	1.275940

5

C	0.000000	0.000000	0.000000
H	-0.569950	-0.928910	0.000000
H	-0.591300	0.914880	0.000000
C	1.371460	0.000000	0.000000
H	1.822050	0.996120	0.000000
Si	2.072170	-1.088340	-1.559790
Si	2.072170	-1.088340	1.559790
H	3.531860	-1.096860	-1.341880
H	3.531860	-1.096860	1.341880
H	1.651940	-0.321390	-2.748430
H	1.651940	-0.321390	2.748430
H	1.428980	-2.412220	-1.444830
H	1.428980	-2.412220	1.444830

6

C	0.000000	0.000000	0.000000
H	0.548720	-0.946500	0.000000
H	0.657000	0.874730	0.000000
Si	-1.893950	0.000000	0.000000
H	-2.436940	1.366210	0.000000
H	-2.103880	-0.831490	-1.224970
H	-2.103880	-0.831490	1.224970

7

C	1.38132	0.00000	0.00000
C	0.00000	0.00000	0.00000
H	-0.55112	0.93484	0.00000
H	1.95078	0.92597	0.00000
C	-0.66830	-1.20884	0.00000
H	-1.75425	-1.25869	0.00000
H	-0.13180	-2.15485	0.00000
H	1.95001	-0.92706	0.00000

8

C	0.00000	0.00000	0.00000
C	1.38903	0.00000	0.00000
C	-0.80474	1.27551	0.00000
H	1.96115	0.92535	0.00000
C	-0.57392	-1.26364	0.00000
H	-1.65229	-1.40210	0.00000
H	0.03234	-2.16521	0.00000
H	1.96086	-0.92373	0.00000

H	-1.87574	1.07501	0.00000
H	-0.57180	1.87710	-0.88186
H	-0.57180	1.87710	0.88186

9

C	0.00000	0.00000	0.00000
C	1.36795	0.00000	0.00000
H	-0.55398	0.93378	-0.00000
H	1.93723	0.92503	0.00000
C	-0.68731	-1.21919	0.00000
C	-2.13336	-1.37694	0.00000
H	-0.08976	-2.13169	0.00000
H	1.93849	-0.92501	0.00000
H	-2.69396	-0.44420	-0.00000
H	-2.42540	-2.00160	0.86089
H	-2.42540	-2.00160	-0.86089

10

C	0.00000	0.00000	0.00000
C	1.35518	0.00000	0.00000
H	-0.55153	0.93578	0.00000
H	1.92352	0.92428	0.00000
C	-0.72127	-1.22389	0.00000
C	-2.12419	-1.35927	0.00000
H	-0.11939	-2.13508	0.00000
H	1.92743	-0.92323	0.00000
H	-2.63793	-0.39210	0.00000
Si	-2.74000	-2.39397	1.57506
Si	-2.74000	-2.39397	-1.57506
H	-4.20393	-2.51518	1.40892
H	-4.20393	-2.51518	-1.40892
H	-2.36466	-1.59009	2.75888
H	-2.36466	-1.59009	-2.75888
H	-2.04018	-3.69795	1.53999
H	-2.04018	-3.69795	-1.53999

11

C	1.36795	0.00000	0.00000
H	1.93793	-0.92443	0.00000
H	1.93818	0.92430	0.00000
C	0.00000	0.00000	0.00000
C	-0.72831	-1.24458	0.00000
C	-2.10134	-1.23394	0.00000
C	-2.78152	-0.00064	0.00000
C	-0.72888	1.24435	0.00000
C	-2.10188	1.23300	0.00000

H	-0.17768	-2.17938	0.00000
H	-2.66327	-2.16030	0.00000
H	-3.86730	-0.00097	0.00000
H	-0.17878	2.17948	0.00000
H	-2.66430	2.15905	0.00000

12

C	0.00000	0.00000	0.00000
C	1.34471	0.00000	0.00000
H	-0.55849	-0.93665	0.00000
H	-0.55862	0.93660	0.00000
C	2.57125	-0.00019	0.00000
H	3.64637	-0.00043	0.00000

13

C	0.00000	0.00000	0.00000
C	1.33580	0.00000	0.00000
H	-0.56209	0.93325	0.00000
H	-0.56250	-0.93297	0.00000
C	2.57225	0.00169	0.00000
C	3.99441	-0.01262	0.00000
H	4.43674	0.98501	0.00000
H	4.34519	-0.58936	-0.87114
H	4.34519	-0.58936	0.87114

14

C	0.00000	0.00000	0.00000
C	1.33761	0.00000	0.00000
H	-0.56137	0.93391	0.00007
H	-0.56137	-0.93386	0.00000
C	2.58015	0.00027	-0.00118
Si	4.46578	-0.04719	0.00235
H	4.94191	1.34666	-0.03494
H	4.74113	-0.77276	1.26521
H	4.75752	-0.84741	-1.20973

15

C	0.00000	0.00000	0.00000
C	1.33252	0.00000	0.00000
H	-0.56367	0.93170	0.00000
H	-0.56463	-0.93101	0.00000
C	2.57400	0.01234	-0.00000
C	3.99794	-0.01997	-0.00000
C	4.61984	1.39202	-0.00000
C	4.43075	-0.83427	-1.27642

C	4.43075	-0.83427	1.27642
H	5.51977	-0.91071	-1.24291
H	5.51977	-0.91071	1.24291
H	4.14637	-0.31469	-2.19174
H	4.14637	-0.31469	2.19174
H	4.00701	-1.83862	-1.28147
H	4.00701	-1.83862	1.28147
H	5.70790	1.30350	-0.00000
H	4.32639	1.95701	0.88645
H	4.32639	1.95701	-0.88645

16

C	0.00000	0.00000	0.00000
C	1.33478	0.00000	0.00000
H	-0.56290	0.93223	0.00000
H	-0.56294	-0.93211	0.00000
C	2.58161	0.00240	0.00000
Si	4.47959	-0.06292	0.00000
C	5.11912	1.69267	0.00000
C	4.85549	-1.03618	-1.57224
C	4.85549	-1.03618	1.57224
H	5.94313	-1.15636	-1.62898
H	5.94313	-1.15636	1.62898
H	4.53445	-0.51260	-2.47491
H	4.53445	-0.51260	2.47491
H	4.40817	-2.03173	-1.56216
H	4.40817	-2.03173	1.56216
H	6.21370	1.68454	0.00000
H	4.79900	2.24783	0.88514
H	4.79900	2.24783	-0.88514

17

C	0.00000	0.00000	0.00000
C	1.33556	0.00000	0.00000
H	-0.56098	-0.93370	-0.00000
H	-0.56521	0.93105	0.00000
C	2.58081	-0.01038	0.00000
Si	4.47558	0.00188	0.00000
C	4.93810	0.89687	1.58822
C	4.93810	0.89687	-1.58822
H	6.03188	0.91909	1.64469
H	6.03188	0.91909	-1.64469
H	4.57243	0.38072	2.47780
H	4.57243	0.38072	-2.47780
H	4.57754	1.92731	1.59962
H	4.57754	1.92731	-1.59962

H	4.84579	-1.42990	-0.00000

18

C	0.00000	0.00000	0.00000
C	1.32550	0.00000	0.00000
H	-0.56259	0.93170	0.00000
H	-0.56837	-0.92785	-0.00000
C	2.57381	0.02250	0.00000
C	3.94582	0.00247	0.00000
H	4.37637	1.01199	0.00000
Si	4.63916	-0.97813	-1.58971
Si	4.63916	-0.97813	1.58971
H	6.10773	-0.88261	-1.44823
H	6.10773	-0.88261	1.44823
H	4.12514	-2.36108	-1.50247
H	4.12514	-2.36108	1.50247
H	4.12163	-0.24779	-2.76586
H	4.12163	-0.24779	2.76586
